# 

**Published:** 1802-06

**Authors:** 


### To CORRESPONDENTS.

Dr. Patterson*'s request will be complied with as soon as possible.* Mr. W*'s communication could not have been admitted without the retrenchments he complains of; and the Editors, in compliance with the opinions of many respectable Correspondents, intend in future to admit no personalities which they think inconsistent with the character of a polished scholar.*

END OF VOL. VII.

### INDEX.

ABERDEEN, defcription of the air of 321

Abernethy, Mr. his cafe of, and obfervations on aneuryfms 97

———, his experiments on perfpirable matter noticed 318

Abingdon, fatality among the poor at, 154, *note*

Ablution, on acetous 337

Abftinence, account of an impofition in a cafe of 190

Acetous ablution, its ufes in typhus and fynochus, &c. 337

Acid, new method of preparing acetic 476

———, defcription of a new 381

Acupuncturation, Mr. Coley's account of 235

Adams, Dr. review of his "Obfervations on the Cancerous Breaft" 82, 163

Addington, Mr. his teftimony in behalf of vaccine inoculation 494

Addrefs to Dr. Jenner from the Suffolk Society of Surgeons 386

——— Marquis Cornwallis on vaccine inoculation 203

Alibert, M. his "Differtation on Fevers" reviewed 376

Allen, Mr. his lectures announced 384

Alfton, Dr. his opinion on the internal virtues of opium 359

Amber, experimental enquiry into the origin of 92

America, on the poifonous ferpents of 478

Anafarca, Mr. Coley's cafe of, with obfervations 406

Aneuryfms, Mr. Abernethy's cafe of 97

Animal bodies, on the influence of chemiftry upon 460

Antichrist, extraordinary opinion concerning 110

Antiloimicus on ablutions in fever 341

Apoplexy, Mr. Davies's cafes of 74

———, Dr. Langflow, in explanation upon 77

———, Mr. Crowfoot's "Obfervations on" reviewed 89

———, Mr. Wilkinfon's remarks on the controverfy concerning 138

———, Dr. Girdleftone's reply to Dr. Langflow on 149

———, Mr. Crowfoot's letter on 150

———, Mr. Atkinfon's remarks on the controverfy between Dr. Langflow and Mr. Crowfoot on 151

———, Dr. Moffman on the fame 206

———, Mr. Davies in explanation 259

———, Dr. Langslow in reply to Mr. Crowfoot 273, and to Mr. Wilkinfon 275, and to Mr. Atkinfon 277

———, Pyrrho on the fame 328, 515

Apoplexy, Mr. Crowfoot in reply to Drs. Moffman and Langflow 361

———, Mr. Atkinfon in reply to Dr. Langflow 363

———, Mr. Hare on the controverfy 397

———, Mr. Wilkinfon in anfwer to Dr. Langflow 436

———, Mr. Atkinfon in anfwer to the fame 441

———, Dr. Langflow's farther obfervations 445

———, Philo-medicus on the nature and cure of 454

———, obfervations on 458

———, Mr. Clement on 513

*Arbor Integra, et Ruinofa Hominis*, extract from 401

Arm prefentation, on delivery in difficult cafes of 481

Arragoni, Dr. inoculated for the cow-pox 182

Ariftarchus, jun. on controverfy 260, his explanation of a query 371

Arfenic, cafe of its ufe in cancer 390

Afafœtida, its ufe in the hooping-cough 506

Afcarides, Dr. Brown on 256

Afphyxia Azotica, obfervations on 511

Atkinfon Mr. his obfervations on apoplexy 151, anfwered by Dr. Langflow 277, his reply 363, his farther obfervations on that fubject 441

Atmofphere, experiments and obfervations on that of marfhes 113

Auguftin, Dr. on the medical ufes of galvanifm (with an engraving) 242

Aufonius, an epigram of 404

Autumn, on the prevalence of typhus in 54

Autumnal Fevers, Dr. Peaal, on 320

Azotic gas, experiments on the action of phofphorus in 374

Babington, Dr. appointed phyfician in ordinary to Guy's Hofpital 384

Baillie, Dr. his remarks on the ftructure of fchirrhus, 85; his conceffion to Dr. Hull, 104; his opinion of tympanites 235

Bark, account of a new preparation of the Peruvian 473

Barnwell, Dr. his fketches of medical geography noticed 319

Bath, lift of difeafes at the difpenfary in 14, 187, 302, 553

———, Mr. Blegborough's defcription of the vapour (with an engraving) 289

Batty, Dr. his lectures announced 95, 191

Beddoes, Dr. his laudable exertions noticed 8

———, on the calx muriata as a remedy for fcrofula 58

Bedford, (duke of) incalculable lofs of 379
Bell, Mr. Benjamin, on tympanites 234
Bentham, Gen. his method of preferving water fweet in long voyages 477
Bergius, on the application of opium to the fkin 125
Bichat, M. his "General Anatomy" reviewed 472
Bifchoff, Dr. on galvanifm and its medical application 529
Blackfmiths, on the deafnefs of 423
Blair, Mr. his lectures announced 95
Blane, Dr. his teftimony before the Committee cf the Houfe of Commons in behalf of vaccine inoculation 489
Blegborough, Mr. on the vapour bath 289
Bluhm, Dr. reftores Kotzebue to health 94
Blumenbach, Prof. a tranflation of his Phyfiology announced 94
Bockman C. W. his "Experiments on the Action of Phofphorus in different Kinds of Gafes" reviewed 374
Bones, on the difeafes of the 415
Booby, Mr. account of 394
Books, medical, critical retrospect of—Kretfchmar, on the Effects of Medicines 80. Prof. Schumacher's Medical and Surgical Obfervations 81. Dr. Adams's Obfervations on the Cancerous Breaft 82, 165. Mr. Crowfoot's Obfervations on Apoplexy 89. Mr. Ring's Treatife on the Cow-Pox 168. Dr. Sacco's Obfervations on the Cow-pox 169. Dr. De Carro's Obfervations on Vaccine Inoculation 185. Prof. Fifcher's Fragments of Natural Hiftory 372. Mr. Nemnich's Lexicon Nofologicum 374. Prof. Hildebrand on the action of Phofphorus ib. M. Alibert's Differtation on Intermittent Fevers 376. Dr. Ontyd's Medical Magazine 377. Cit. Richerand's New Elements of Phyfiology 469. Cit. Hubert on Oxygen Gas on Germination 470. Cit. Hauy's Treatife of Mineralogy 471. Mr. Bichat's General Anatomy 472. Thefaurus Medicamentum, or a New Collection of Medical Prefcriptions, &c. 564. A Practical Synopfis of the Materia Medica, Vol. ii. Part I. 565
Bougies, examination of the metallic 238
Bradley, Dr. his lectures announced 95
———, his teftimony in favor of vaccine inoculation 491
Breaft, on fchirrhous tumour in the 49
———, on cancer in the 82, 163
Brown, Dr. on afcarides 256
Briftol, lift of difeafes at the difpenfary of 14, 304
——— Hotwells, account of difeafes at the Pneumatic Inftitution at the 301
Britain, etymology of the word 240
Bruant Cit. on the ophthalmia of Egypt 212
Brunonian champions, violent zeal of the 93
——— practice, curious inftance of 407
Bullet Forceps, defcription of an improved 554
Cadiz, account of the peftilential fever at 313
Calculi, on the generation of urinary 64
Callifen on the Intus-Sufceptio 38
Calomel, experiments on 546
Caloric, fluids non-conductors of 480
Calx muriata, on the ufe of 58
Cam, Mr. his cafe of monftrofity, (with a plate) 385
Cancer, Dr. M. Whiiter's cafe of 52
———, Mr. Cuftance's cafe of 390
Catalepfy, Dr. Maton's cafe of 30
———, Dr. Lubbock's cafe of 105
Cataract, Mr. Crowfoot's cafe of 404
Catarrhus fuffocativus, cafes of the 387
Celfus, his directions for treating the tympany 230
Chalk, its ufe in cancers 51
Chamberlaine, Mr. on medical opinions in courts of juftice 283
Chaptal on the air in the Mediterranean 123
Chemiftry, its influence on animal bodies 461
Chenevix, Mr. his differtation upon chemical nomenclature announced 239, *note*
———, Mr. his experiments on the metallic combinations of muriatic acid 546
Chefelden, his cafe of umbilical hernia 35
Chevalier's Mr. lectures announced 95
———, cafes of diffection 387
———, his defcription of an improved bullet forceps 554
Ching-Si, the quack 396
Cholera-Morbus, opium recommended in 305
Clarke's Dr. lectures announced 95
Clark's examples quoted on the plague of London 311
Clement, Mr. on apoplexy 513
Cline, Mr. on cancerous tumours 87
———, his teftimony before the Committee of the Houfe of Commons in behalf of vaccine inoculation 496
Cogan, Dr. on vaccine inoculation 251
Colchefter Medical Society, their teftimonial in behalf of vaccine inoculation 528
Cole, Mr. his cafe of tetanus 55
Coley's, Mr. cafe of tympanites 223
———, on acupuncturation 234
———, cafe of anafarca, &c. 406
Collyria, forms of 215
Columbium, a new metal difcovered of that name 477
Committee of the Houfe of Commons, their report on vaccine inoculation 485
Contagion, on the means of correcting 424
Cooke, Mr. on contagious fever 255
Cornwallis, addrefs to Marquis 201
Corfica, culture of exotics in 479
Correfpondents, notes to, 96, 192, 288, 384, 480
Corrigenda 191

Cow-pox, Mr. Fuge's letter to Mr. Dunning on 7

———, Dr. Stevenson's communication on the spurious or imitative 9

———, Review of Mr. Ring's "Treatise on the" 168

———, of Dr. Sacco's "Observations on the" 169

———, Dr. Winterbottom, on the 294

———, opinion of an American physician on, 317, with a note by Dr. Paterson

Crocodiles, on the difference between those of the antient and those of the new world 575

Crowthers', Messrs. method of treating compound fractures 307

Crowfoot, Mr. review of his "Observations on Apoplexy" 89

———, his remarks thereon 150

———, his answer to Drs. Moffman and Langslow 361

———, his case of and observations on the cataract 405

Cruickshank, Mr. his death occasioned by apoplexy, 77, *note*

———, his chemical system replied to 380

Crump, Dr. his opinions on opium, controverted, 128, *et seq.*

Cullen, Dr. his description on the tympanites 232

———, on narcotic sedatives, 127, *note*

Curry, Dr. elected assistant physician to Guy's Hospital 384

Custance, Mr. on the use of arsenic in cancer 390

Cuvier, Cit. a translation of his comparative anatomy announced 94

———, Cit. on the true difference between the crocodiles of the antient and new world 575

Darwin, Dr. his description of tympanites 234

Davies, Mr. his cases of apoplexy 74

———, his letter of explanation 259

———, on the cure of Pertussis 506

Deafness, on that of blacksmiths 423

De Carro, Dr. extract from his letter to Mr. Ring on the cow-pox 110

———, his observations on vaccine inoculation, reviewed 185

Degrees, Philo-Medicus on granting medical 12, 161, 366

Denman's, Dr. description of the hydrops vagina 411

Dennison's, Dr. lectures announced 95

Deveze, Mr. on effusions under the skull 216

Diabetes, on the powers of lime-water in the 66

———, Dr. Lubbock's cases of 107

———, on the use of opium in, 504, *note*

Dieman's, Dr. idea of power disputed 461

Dimsdale, Baron, exposes the Suttonian mode of inoculation 496

Digitalis, Mr. Gapper on the uses of 155

———, Dr. Maclean's remark on its use, in discharging serous fluids 197

———, its success in cases of tympanites 228

———, its inefficiency in hydrothorax 401

Diseases, in the Bath Dispensary, list of 14, 187, 302, 553

———, in the Bristol Dispensary 14, 304

———, in an eastern district of London, from the 20th of November to the 20th of December, 1801 15

———, from the 20th of December, 1801, to the 20th of January, 1802 111-12

———, from the 20th of January, to the 20th of February, 1802 287-8

———, from February 20th, to March 20th 403-4

———, from March 20th to April 20th 468

———, from April 20, to May 20, 1802 556

———, in the Westminster Hospital, from November 26th to December 26th, 1801 15, 16

———, of the year 1801 112

———, at the Pneumatic Institution, Bristol Hotwells 301

Dissection, cases of 387

Docker, Mr. on opiate frictions 258

Dogs, result of inoculating them with vaccine virus 179

Dolœus, his recipe in fevers 404

Domestic quackery, on 392

Dropsy, on the use of mercury in 401

———, Mr. Coley's case of 407

Druggists, impropriety of applying to them to make up prescriptions 370-1

Dunning, Mr. his case of premature menstruation 1

———, on vaccination 2, 191

Dyspepsia, on the method of treating 425

Edinburgh, account of medical education at 42

Effusion, on treating that under the scull 216

Egypt, on the ophthalmia of 212

Electricity, on animal 160

———, remarks on 324

———, Mr. Nicholson's conclusions from experiments in 478

Elgin, Lord, corresponds with Dr. De Carro on vaccination 186

Emigration, shocking descriptions of Irish 381

Epidemic diseases, on publishing accounts of 285

Errata 288, 384

Exfoliation, case of 23

Exotics, account of their culture in Corsica 479

Experiments on the atmosphere of marshes by Dr. Seybert 113

———, Mr. Chenevix's on the metallic combinations of muriatic acid 546

———, Cit. Prevost's, on odorous substances 572

———, on opium, by Mr. Ward 124, 341, 497

———, on the metallic bougies, by Mr. Vandier 238

———, Dr. Augustin's galvanic 242

———, on the action of phosphorus in different gases 374

———, Dr. Priestley's on the pile of Volta 381

———, on the influence of air, &c. on germination 470

———, on the constituent particles of urine 474

———, Galvanic 533

Eye, on inflammations of the 209

Farquhar, Sir Walter, his testimony before the Committee of the House of Commons in behalf of vaccine inoculation 492

Farr's Dr. medical jurisprudence recommended 283

Ferguson, Dr. on the deafness of blacksmiths 423

Fevers, on the treatment of 38

———, on contagious 255

———, on pestilential 320

———, review of "Alibert's Dissertation on" 376

Fischer's, Prof. "Fragments of Natural History" reviewed 372

Fluids, account of Dr. Murray's experiments on 480

Fordyce, Dr. G. his opinion on apoplexy 77

Fothergill, Dr. his mistake on apoplexy accounted for 208

———, his case of apoplexy 442

Fourcroy, Cit on the urinary stones and gravel in men 60

Fracture, Dr. Girdlestone's case of 53

———, account of a successful mode of treating compound 307

Friction, Mr. Docker on opiate 258

Frogs, Salvani's experiments on 71, 159

———, Mr. Volta's experiments on 160

———, the effects of opium on 124, 359

Fuge Mr. his letter to Mr. Dunning on the cow-pox 7

Galvanic discovery, historical statement of the 70, 159

Galvanism, on the medical use of (with a plate) 242

———, Dr. Bischoff on (with plates) 529

Gapper Mr. on the effects of digitalis in rheumatism 155

Garnet's, Dr. lectures announced 94

Gases, experiments on different kinds of 374

Germany, success of the vaccine inoculation in 171

Germination, the influence of air and other substances on 470

Girdlestone, Dr. his case of fracture 53

———, on typhus fevers in autumn 54

———, on apoplexy, in reply to Dr. Langslow 149

———, on the use of mercury in dropsy 401

Girtanner, Mr. his opinion concerning amber 92

Goettling, Mr. on his assertion that phosphorus shines in azotic gas 374

Gonorrhœa, on the use of tinct. nicotiana in the 57

Gremb's "*Arbor Integra et Ruinosa Hominis*" quoted 401

Guy's Hospital, elections in the Physical Society of 94

Hamilton, Dr. his letter on the vapour bath 291

Hare, Mr. on apoplexy 397

Hat, its inconvenience in a hot climate 215

Hatchett, Mr. his discovery of a new metal 477

Hauy, Cit. "*Traité de Minéralogie*" reviewed 471

Haygarth, Dr. his regulations to prevent the spread of small-pox recommended 3

Heister, his account of the tympany 231

Hermbsted, Prof. his experiments on amber 92

Hernia, case of umbilical 35

———, necessity of more attention being paid to 379

Herodotus, his remark on the difference between the head of an Egyptian and that of a Parthian 215

Hey, Mr. his "Observations in Surgery" announced 480

Hill, Mr. on the diseases of the bones 415

Hippocrates, his axiom on apoplexy 337

Historical statement of the galvanic discovery 70, 159

Hodson's, Mr. new mode of extracting a stone 429

Hoffman, his opinion on the tympanites 231

Hooper's "Medical Dictionary" quoted 126

Hooping cough, on the use of opium in the 305

———, on the cure of 506

Huber, Cit. his experiments on the influence of gases on germination 470

Hull, Dr. on intus susceptio 32

Hunter, Dr. on the hydatid 83

Hydatid, description of the human ib.

Hydrocephalus, why incurable 207

Hydrops pulmonum, case of 407

Hydrothorax, observations on 401

Inflammations, Dr. White's mode of managing 209

———, postscript to that paper 212

Ingenhousz, on aquatic plants 123

Inoculation, vaccine, Mr. Dunning on 2

———, Mr. Fuge on 7

———, on rewarding the discoverer of 11

———, Mr. Ring on 109, 292

———, Dr. De Carro's observations on 185

———, Mr. Moor's communication on 200

———, French address to Marquis Cornwallis on 201

———, Mr. Pears on 249

———, Dr. Cogan on 251

———, Suffolk surgeons testimonial in favour of 386

———, experiments on at Rotterdam 252

———, report of the Committee of the House of Commons on 485

———, Dr. Blane's testimonial on 489

———, Dr. Bradley's ditto 491

———, Dr. Woodville's ditto ib.

———, Sir Walter Farquhar's ditto 492

———, Mr. Ring's ditto ib.

———, Dr. Sims's ditto 493

———, Mr. J. Addington's ditto 494

———, Dr. Lettsom's ditto ib.

———, Mr. Cline's ditto 496

———, the Colchester Medical Society's ditto 528

———, Mr. Wales's letter on 540

Intelligence, medical and physical 92, 190, 384, 481

Intermittent fevers, " Dissertation on" reviewed 376

Intestine, case of sphacelation of the 430

Intus-susceptio, observations on the 32

Inversio-uteri, two cases of 433

Irish emigration, dreadful accounts of 381, 383

Itch, on the treatment of the 293

Jackson's, Dr. case of sphacelation of the intestine 430

Jameson R. discovers a new acid 381

Jameson's, Dr. lectures announced 575

Japanese, account of their operations in tympany 236

Jenner, Dr. proposals for rewarding 11, 378

———, notice of Mr. Northcote's portrait of 95

———, Dr. De Carro's opinion of 110

———, Dr. Sacco's encomium on 170

———, French address to Marquis Cornwallis concerning 201

———, testimonial of the Suffolk Society of Surgeons addressed to 386

———, report of the Committee of the House of Commons on the petition of 485

———, Mr. on spurious vaccine pustule 203

Johnson, Dr. Samuel, anecdote of 162

Johnstone, Dr. on the use of vinegar in preventing contagion 424

Juncker's opinion of the tympanites 231

Junius, on the letters of 369

Kant, his opinion of power 461

Kine-pox, introduced into America 316

King, Mr. his account of the Pneumatic Institution at Bristol 301

Kinglake, Dr. on dyspepsia 425

———, on sedative efficiency 522

Kirkland, Dr. his reasoning on apoplexy controverted 90

Knight, Mr. his proposal for a medical memorial in honour of Dr. Jenner 378

Kotzebue, false account of his illness 93

Kretschmar, Fred. review of his " Essay on the Effects of Medicines" 80

Lagrange's chemistry, account of a new edition of 480

Langslow, Dr. in explanation upon apoplexy 77

———, his reply to Dr. Girdlestone, Messrs. Crowfoot, Wilkinson, and Atkinson, on apoplexy 273, 283

———, his farther reply on the same subject 445, 454

Lavoisier's analysis of animal and vegetable substances 119

Lead, observations on the sugar of 507

LECTURES ANNOUNCED. — Dr. Garnett's on chemistry, 94, on ZOONOMIA, 95 — Dr. BRADLEY's on the theory and practice of medicine, ib. — Dr. BATTY's on the theory and practice of midwifery, ib. — On ditto, and the diseases of women and children, 191, 384 — Dr. DENNISON and Dr. SQUIRE on the same, 95, 384 — Dr. OSBORN's and Dr. CLARKE's on the same, ib. — Mr. BLAIR's course of clinical lectures, ib. — Mr. CHEVALIER's on the principles and operations of surgery, ib. — Dr. POLE and Mr. WAGSTAFFE on the theory and practice of midwifery, and the diseases of women and children, 191 — Mr. MACARTNEY on comparative anatomy and physiology, ib. — Mr. THOMAS on surgery, ib. — Mr. ALLEN on the animal economy, 384 — Dr. JAMESON's clinical on the principles and practice of medicine, 575.

Lee, Mr. his refutation of Dr. Mitchill's hypothesis on nitric acid 317

Lettsom, Dr. his testimony before the House of Commons in behalf of vaccine inoculation 494

Lime-water, its sanative powers in diabetes 66, 108

Lind, on the application of opium in tetanus 125

Locke, Mr. on the use of words 10

Locusts, visit America in 1800 319

Lombardy, cow-pox discovered in 172

London, anniverſary of the Medical Society of 297

———, account of the plague of 311, *note*

Londonderry, ſketch of the weather at 509

Lubbock's, Dr. caſe of catalepſy 105

———, caſe of diabetes 107

Lucas, Mr. his caſe of a ſchirrhous tumour in the breaſt 49

Macartney's, Mr. lectures announced 191

Maclean, Dr. on tapping during pregnancy 193

M'Whiſter's, Dr. caſe of cancer 51

Marſhall, Dr. his miſſion to ſpread vaccination 480

Marshes, on the atmoſphere of 113

Maton's, Dr. caſe of catalepſy 30

Meaſles imported into America 316

Mead, Dr. on the tympany 232

Medals propoſed by the London Medical Society 300

Medical-degrees, Philo Medicus on, 13, 161, 366

——— Education at Edinburgh, account of, 42

——— and phyſical intelligence, 92, 190, 384, 481

——— and Surgical Obſervations, review of 81

——— Publications new, liſt of 96

——— critical retroſpect of books v. Books

——— Society, London Medical, anniverſary meeting of the 297

———, officers and council of 299

———, prize-medals offered by 300

Medicines, review of " An Eſſay on the Effects of" 80

Medicus on domeſtic quackery 392

Menſtruation, case of premature 1

Mercury, its uſe in rheumatiſm, 315, *note*

———, its uſe in dropſy 401

Metallic bougies, experiments on the 238

Mineralogy, review of a treatiſe of 471

Mirbel, Cit. his obſervations on vegetable anatomy 557

Mitchill, Dr. his hypotheſis on nitric acid controverted 317

Moiſes, Dr. his caſe of needles, &c. extracted 17

———, on acetous ablution 337

Monro, Dr. his caſe of inverted inteſtine 37

———, on the effect of opium on frogs, 124

Monſtroſity, caſe of 385

Montroſe, Ducheſs of, expoſes an empyrical impoſture 395

Moore, Mr. on vaccine inoculation 200

Moſſman, Dr. on apoplexy 205

Muriatic acid, metallic combinations of 546

Murray, Dr. his experiments on fluids noticed 480

*Naturhiſtoriche Fragmente*, review of 372

Needles, &c. extraordinary caſe of the extraction of 17

Negroes, their thick ſkins accounted for 215

Nervous fluids, on the identity of 324

Nemnich's *Lexicon Noſologicum*, &c. reviewed 374

New York, population of, increaſed 320

Nicholſon's, Mr. concluſions from electrical experiments 478

Nicotiana ſucceſsfully employed in Gonorrhœa 57

Nitric Acid, refutation of Dr. Mitchill's hypotheſis on 317

Noiſette, Cit. his ſucceſs in the culture of exotics in Corſica 479

Northcote, Mr. notice of his picture of Dr. Jenner 95

Ocular inflammations, obſervations on 209

Odontagra, deſcription of Mr. Reece's 220

Odorous ſubſtances, on their movements on the ſurface of water 572

Officers and council of the Medical Society of London, for 1802 299

Old Practitioner, his reply to Philo-Medicus on medical degrees 271

Ontyd Dr. his Medical Magazine reviewed, 377

———, on the influence of chemistry upon animal bodies 261, 460

Ophthalmia, on that of Egypt 212

Opiate frictions, Mr. Docker on 258

——— recommended in phthiſis pulmonalis 503, *note*

Opinions, on thoſe given by medical men in courts of juſtice 283

Opium, its ſucceſs in a caſe of tetanus 55

———, obſervations on the modus operandi of 124, 341, 497

Orang Outang, obſervations on the 371

Oſborn's, Dr. lectures announced 95

Oxyd, account of an acidifiable 477

Oxygen gas, its effects on germination 470

Paracenteſis, caſe of tympanites relieved by the operation of 223

Parey, Ambroſe, quoted 230

Paris, bad ſtate of the water at 477

Patterſon, Dr. his communication of a letter to him from Philadelphia 311

———, his ſketch of the weather at Londonderry in 1801 507

———, his communication to Dr. Bradley on aſphyxia azoticæ 511

Peaal's, Dr. obſervations on the autumnal fever at Aberdeen 321

Pears, Mr. on vaccine inoculation 249

Pertuſſis, on the cure of 506

Peruvian bark, new preparation of 473

Peterſburgh, ſucceſs of the vaccine inoculation at 109

Philadelphia, increaſe of the population of 320

Philo-Medicus, on medical graduation, 13, 161, 366

———, on the nature and cure of apoplexy 454

——— Londinenſis, on publiſhing accounts of epidemic diſeaſes 285

Phosphorus, its action in various kinds of gases 374

Physiology, new Elements of, reviewed 469

Piguillem, Dr. a Spanish physician, practises the vaccine inoculation 171

Pile, observations on Volta's 381

Pins, extraordinary case of the vomiting of, 20

Plant, account of a singular American, 479

Plenck, on the principles of cutaneous perspirable matter 319

Pneumatic Institution, list of diseases at the Bristol 301

Poison, account of a plant which repels that of serpents 479

Pole, Dr. his lectures announced 191

Poor, their great distress 155

Practical Synopsis of the Materia Medica, review of 565

Pregnancy, on tapping during 40, 193

Prevost, Cit. on the movements of odorous substances on water 572

Priestley, Dr. his reply to Mr. Cruickshank, 380

———, his observations on the pile of Volta 381

Pringle, Sir John, his opinion on tympany 232

Prize Medals, list of those given by the London Medical Society 300

Proust, Mr. his experiments on the composition of urine 474

Psora, observations on 520

Purton's, Mr. remarks on electricity 324

Pyrrho, on apoplexy 328, 515

Quack advertisements, impudence of 396

Quackery, causes of 370

———, on domestic 392

Ramsay, Dr. notice of his oration on the improvements in medicine in the course of the eighteenth century 319
Reece, Mr. description of his Odontagra, or instrument for extracting teeth (with an engraving) 220
———, acknowledgement to 390
———, his new method of preparing bark 473
Report of the Committee of the House of Commons on vaccine inoculation 485
Rheumatism, virtues of the digitalis in the 156
———, use of mercury in 315, *note.*
Richerand's Cit. "New Elements of Physiology," reviewed 469
Ring, Mr. on vaccine inoculation 107, 292
———, his "Treatise on the Cow-Pox" reviewed 168
———, on the treatment of the itch 293
———, his testimony in behalf of vaccine inoculation 492
Rotterdam, account of the vaccine inoculation at 253
Royal College of Physicians at London, their judicious regulation 47
Rumford, Count, his opinion on caloric controverted 480
Russia, progress of vaccine inoculation in 292
Ryan's, Mr. observations on the acetite of lead 507

Sacco, Dr. of Milan, communicates vaccine matter to Dr. Jenner 109

———, his observations on the cow-pox reviewed (with plates) 169

Savaresi, Dr. his observations on the Egyptian Ophthalmia, objections to 213

Scherer's Chemical Journal quoted 381

Schmidtman, Dr. his account of a singular imposture 190

Schumacher, Prof. his "Medical and Surgical Observations" reviewed 81

Schutz, Dr. on the sanative powers of lime-water in the diabetes 66

Scirrhous tumour, case of one in the breast, 49

——— Structure, Dr. Baillie, on 85

Scrofula, inefficiency of calx muriata in 58

Scull, case of a fracture of the 53

———, on effusions under the 219

Searchers, their ignorance noted 251

Sedatives, definition of 126

———, Dr. Kinglake, on 522

Serpents, account of a plant that repels the poison of 479

Seton, its use in Ophthalmia 215

Seybert's, Dr. experiments and observations on the atmosphere of marshes 113

Sheep, result of inoculating them with vaccine virus 241

———, description of a particular disorder in 241

Simmons, Mr. on the use of tinct. nicotianæ 57

———, on the calx muriata in Scrofula 58

Sims, Dr. account of his speech at the anniversary meeting of the London Medical Society 297

———, on delivery in certain difficult cases of arm-presentation 481

———, his testimony to the Committee of the House of Commons in favour of vaccine inoculation 493

Smallpox, Dr. Haygarth's regulations for preventing recommended 3

———, decrease of its mortality in 1801 112

Smith's metallic bougies, examination of 238

Spain, progress of vaccine inoculation in 171

Sphacelation of the intestine, case of 430

Spurious vaccine pustule, remarks on 203

———, Dr. Sacco on 184

Squire's, Dr. lectures announced 95

Stevenson Dr. on the spurious vaccine 9

Stimulants, definition of 126

Stone, new mode of extracting the 429

Stone and gravel in men, Cit. Fourcoy on 60

Suffolk, addrefs to Dr. Jenner from the Society of Surgeons in the county of 386
Sulzer's Theory of Senfations, quoted 70

Tapping during pregnancy, Dr. Vieuffeux on 40
———, Dr. Maclean on 193
Tatham and Egg, account of their new invented trufs 380
Teeth, defcription of an inftrument for extracting (with an engraving) 220
Tetanus, ca.e of 55
———, on the application of opium in 125
Thefaurus Medicamentum, review of the 564
Thomas's, Mr. lectures announced 191
Tinea capitis, efficacious remedy for 293
Trotter, Dr. prefented by the furgeons of the fleet with a piece of plate 576
Trufs, account of a newly invented 380
Tulpius, on premature menftruation 2
Turkey, progrefs of vaccine inoculation in 292
Turks, Baron de Tott's defcription of the 135
Turner, Mr. Francis, his memory refpected 54
Typhus, on the prevalence of in autumn 594

Univerfities, comparifon between Edinburgh and the Englifh 48

Urinary ftones and gravel in men, Cit. Fourcroy on 60

Urine, experiments on the compofition of 474

Vaccination, Mr. Dunning on 1, 191
Vaccine inoculation, Mr. Fuge on 7
———, D. T. on 11
———, Mr. Ring on 109, 292
———, Dr. De Carro's obfervations on 185
———, Mr. Moore on 200
———, French addrefs to Marquis Cornwallis on 201
———, Mr. Pears on 249
———, Dr. Cogan on 251
———, Mr. Knight on 378
———, Suffolk Society of Surgeons on 386
———, Report of the Committee of the Houfe of Commons on 485
———, Teftimonial of the Colchefter Medical Society in favor of 528
———, Mr. Wales on 540
Vaccine puftule, on the fpurious 203
Vage, Dr. his criticifms on the treatment of the venereal difeafe 559
Vandier, Mr. his experiments on metallic bougies 238
Vangas, Don Pedro, his experiments on ferpents 478
Van Swieten, his account of the tympanites 230
Vapour bath, defcription of the (with a plate) 289
Vegetable acids, their ufes in difeafes 337
Vegetable anatomy, obfervations on 557
Venereal difeafe, criticifms on the treatment of 559
Vieuffeux, Dr. on tapping during pregnancy 40
Vinegar, its ufes in fevers 39, 255, 341
———, new method of preparing radical 476
Volonlus, obfervations on the 32
Volta, on the pile of by Dr. Auguftin 242
———, Dr. Prieftley's experiments on 381
Vomits, injudicious in apoplexy 76, 78, 208
———, their ufe defended 329, 398

Wales, Mr. on vaccine inoculation 540
War, the miferies brought on the poor by the 153
Ward, Mr. his experiments on opium 124, 341, 497
Warner, Mr. on the benefit of opium in the hooping cough 305
Water, new method of preferving it during long voyages 477
———, mode of filtering at Paris ib.
———, on the movements of odorous fubftances on the furface of 572
——— flea, defcription of the 191
Watkinfon, Mr. on the inverfio uteri 433
Webfter, his opinion on the caufe of the yellow fever ridiculed 312, *note*
Weftminfter Hofpital, vide, *Difeafes in the Weftminfter Hofpital*
White, Mr. his explanation refpecting the bilious diarrhœa 188
Whitworth doctor, his practice 395, *note*
Whyte, Dr. on inflammations of the eye 209
Whytt, on the external ufe of laudanum 125
Wilkinfon, Mr. on apoplexy 138
———, his reply to Dr. Langflow 436
Wilfon, Dr. on opium quoted 344
Winterbottom, Dr. on the cow-pox 294
Withering, Dr. his opinion on the effects of digitalis in hydrothorax 401
Wood, analyfis of bituminous 381
Woodville Dr. his teftimony before the Committee of the Houfe of Commons in favour of vaccine inoculation 491

Yearly bill of mortality, 1801 112
Yellow fever, imported into Cadiz from America 313

Zurine, Mr. his remarks on the water flea 191

*References*

### *References to the Plates.*

| | |
|---|---|
| The Appearance of the Vaccine Puftules on the Udder of a Cow | 185 |
| Galvanic Apparatus and Mr. Reece's Odontagra - - | 242 |
| Smith's Air Pump Vapour Bath - - - - - | 289 |
| Cafe of Monftrofity - - - - - - - - | 385 |
| Dr. Bischoff's Galvanic Apparatus - - - - - | 529 |
| Mr. Chevalier's Bullet Forceps - - - - - - | 555 |

---

New BOOKS lately publifhed by R. PHILLIPS.

In One thick Volume, 12mo. price 6s. in boards, or 6s. 6d bound; containing more Letter Prefs than any other Medical Work of a general and popular nature.

### 1. THE FAMILY PHYSICIAN,

### Or DOMESTIC MEDICAL FRIEND,

Containing plain and practical Inftructions for the Prevention and Cure of Difeafes, according to the laft improvements and difcoveries, with a feries of chapters on collateral fubjects, comprizing every thing relative to the Theory and Principles of the Medical Art neceffary to be known by the private Practitioner; the whole divefted of fcientific and technical words and phrafes, and adapted to the ufe of thofe Heads of Families who have not had a Claffical or Medical Education.

By ALEXANDER THOMSON, M. D.

Author of Medical Confultations; of an Effay on the Difeafes of Women; and of a Treatife on the Nerves.

In One Volume, duodecimo, price 6s. in boards,

### 2. A PRACTICAL TREATISE ON DIET,

And on the moft falutary and agreeable Means of fupporting LIFE and HEALTH by ALIMENT and REGIMEN. Adapted to the various Circumftances of Age, Conftitution, and Climate; and including the Application of modern Chemiftry to the culinary Preparation of Food.

By WILLIAM NISBET, M. D.

Fellow of the Royal College of Surgeons, Edinburgh; Author of the Clinical Guide, &c.

*Critical Commendations of this Work.*

"We are convinced that this compendious Work, on the important Subject of Diet, will prove a valuable and acceptable prefent to the Public."

*Medical and Phyfical Journal.*

"Dr. Nisbet is one of the few Medical Writers among our Contemporaries, who know how to unite Science with lucid and infinuating Simplicity of Manner; the Formalities of Science are avoided, but its Spirit is happily diffufed through the Whole. In his prefent Work he has performed a Service, which deferves the Thanks of every good Man zealous of Public Utility. No Family ought to neglect the Perufal of a Book fo agreeable, and fo interefting inftructive." *London Medical Review.*

"Dr. Nisbet profeffes, that he has collected together a greater Mafs of Matter on the Subject of Diet, than has appeared before in any fingle Book; and we can allow our Author's Claim, for he has collected few Trifles, and has in general diftinguifhed the ufeful Inftruction from fanciful Refinement or fuperftitious Abfurdity. On the whole, we think Dr. Nisbet's Syftem judicious. The Dietetic Chemiftry is an able and fcientific Abftract of the Effects of Cookery on the Subftances employed as Food."

*Critical Review.*

THE SAVAGE OF AVEYRON.

This Day is publifhed, Price 3s. 6d. in Boards, embellifhed with his Portrait;

### 3. AN HISTORICAL AND PHILOSOPHICAL ACCOUNT

Of the Difcovery and Education of A SAVAGE MAN; or, of the firft Developments, Phyfical and Moral, of the YOUNG SAVAGE caught in the Woods, in the Department of Aveyron, in the Year 1798, and brought to Paris, by Order of the French Government, in 1799.

By E. M. ITARD,

Phyfician to the National Inftitution for the Deaf and Dumb, Member of the Medical Society of Paris, &c.

*In a fmall Pocket Volume, Price Five Shillings bound in Red, illuftrated with a Map of all the Roads between London and Paris; with a Plan of Paris; with a Map of the French Republic; and feveral other Copper-plates.*

### 4. THE ENGLISHMAN's COMPANION IN A JOURNEY TO PARIS,

CONTAINING:

1. An ITINERARY of all the great Roads with a Variety of practical Obfervations.
2. A correct Defcription of all the Objects of Curiofity, the Libraries, Mufeums, Buildings, Exhibitions, Publick Amufements, &c. &c. in MODERN PARIS and its environs; with the Terms and Manner in which they may be feen.
3. TABLES of Money, Weights, Meafures, Prices of Provifions, and other neceffary Particulars.
4. INSTRUCTIONS to be obferved upon the Road, and during the Refidence at Paris with Accounts of Inns, Hotels, Coffee-houfes, &c. &c.

In two Volumes, royal 18mo. with Engravings, and a correct Map, price 10s. in boards 11s. half-bound, or 12s. handfomely bound and lettered.

### 5. A HISTORY of GREECE from the EARLIEST PERIODS,

TILL ITS REDUCTION INTO A ROMAN PROVINCE.

Intended principally for the Ufe of Schools, and Young Perfons of both Sexes.

By WILLIAM MAVOR, LL. D.

Vicar of Hurley, in Berkfhire, Chaplain to the Earl of Dumfries, Author of the Britifh Nepos, Univerfal Hiftory, &c.

Of whom may be had Dr. MAVOR's UNIVERSAL HISTORY, in Twenty-five Volume, as each Volume appears on the Firft Day of every Month.

### 6. KOTZEBUE'S EXILE INTO SIBERIA.

In three Volumes, Foolfcap 8vo. Price 15s. in Boards, embellifhed with Engravings,

THE MOST REMARKABLE YEAR IN THE LIFE OF AUGUSTUS VON KOTZEBUE,

Containing a full Account of his late Exile and Journey into Siberia, and of the other extraordinary Incidents which happened to him in Ruffia, with Authentic Particulars relative to the prefent State of the Interior of the Ruffian Empire, and new Anecdotes of the Court of Peterfburgh.

WRITTEN BY HIMSELF.

Tranflated from the German, under the fuperintendance of the Author,

By the Rev. B. BERESFORD, P. D.

Englifh Lecturer to the Queen of Pruffia.

---

[ W. THORNE, Printer, Red Lion Court, Fleet Street. ]

